# Coexistence of intervertebral disc herniation with intradural schwannoma in a lumbar segment: a case report

**DOI:** 10.1186/s12957-016-0864-y

**Published:** 2016-04-18

**Authors:** Jianjiang Pan, Yue Wang, Yazeng Huang

**Affiliations:** Spine Lab, Department of Orthopedic Surgery, The 1st Affiliated Hospital of Zhejiang University, 79# Qingchun Road, Hangzhou, 310003 China; Department of Orthopedic Surgery, Zhejiang Provincial People’s Hospital, 158# Shangtang Road, Hangzhou, 310014 China

**Keywords:** Lumbar disc herniation, Intraspinal tumor, Schwannoma

## Abstract

**Background:**

Lumbar intervertebral disc herniation and spinal tumor are major pathologies that may cause back pain and radiculopathy. Neurological symptoms resulting from disc herniation and intradural spinal tumor together, however, are very rare.

**Case presentation:**

We report a case of lumbar disc herniation which coexists with intradural schwannoma at the same spinal level in a 67-year-old man. The patient presented with persistent low back pain, sciatica, and weakness of the lower limbs. Contrast lumbar spine magnetic resonance (MR) imaging clearly delineated an intradural lesion and an extradural herniated disc at L3/4 level. Using a single posterior approach, both pathologies were addressed. Pathological studies confirmed the intradural lesion was schwannoma.

**Conclusion:**

The case report highlights a rare concomitance of two symptomatic pathologies in a lumbar spine, which deserves clinical attention. Complete history, careful physical examination, and investigative measures, such as contrast MR imaging, are helpful to establish throughout diagnoses.

## Background

Lumbar intervertebral disc herniation is one of the most common spinal disorders that cause back pain and radiating leg pain [1]. Spinal tumor is another category of major pathologies that may lead to pain and neurological symptoms [2]. Occasionally, extradural or intradural tumor may present clinical symptoms similar to those of disc herniation, with confusing image findings. It is, however, rare for the two pathologies to present at the same spinal level. We reported such a case of L3/4 disc herniation coexisting with intradural schwannoma.

## Case presentation

A 67-year-old man presented with low back pain, sciatica, and weakness of the lower limbs for 2 years. His back and leg pain exacerbated after standing or walking and relieved by lying supine with the knees and hips flexed. Overall, his pain was tolerable. He can walk well, though he felt his legs were weaker than usual. He sought treatment at a local hospital, and magnetic resonance (MR) imaging revealed a herniation disc at L3/4 level. Two months ago, however, his back pain worsened considerably and he started to have intermittent claudication. His symptoms failed to respond to conservative treatments, and his walking distance decreased to approximately 100 m. He was referred to us for further treatment. His bowel and bladder functions were normal since he was sick.

**Fig. 1:**
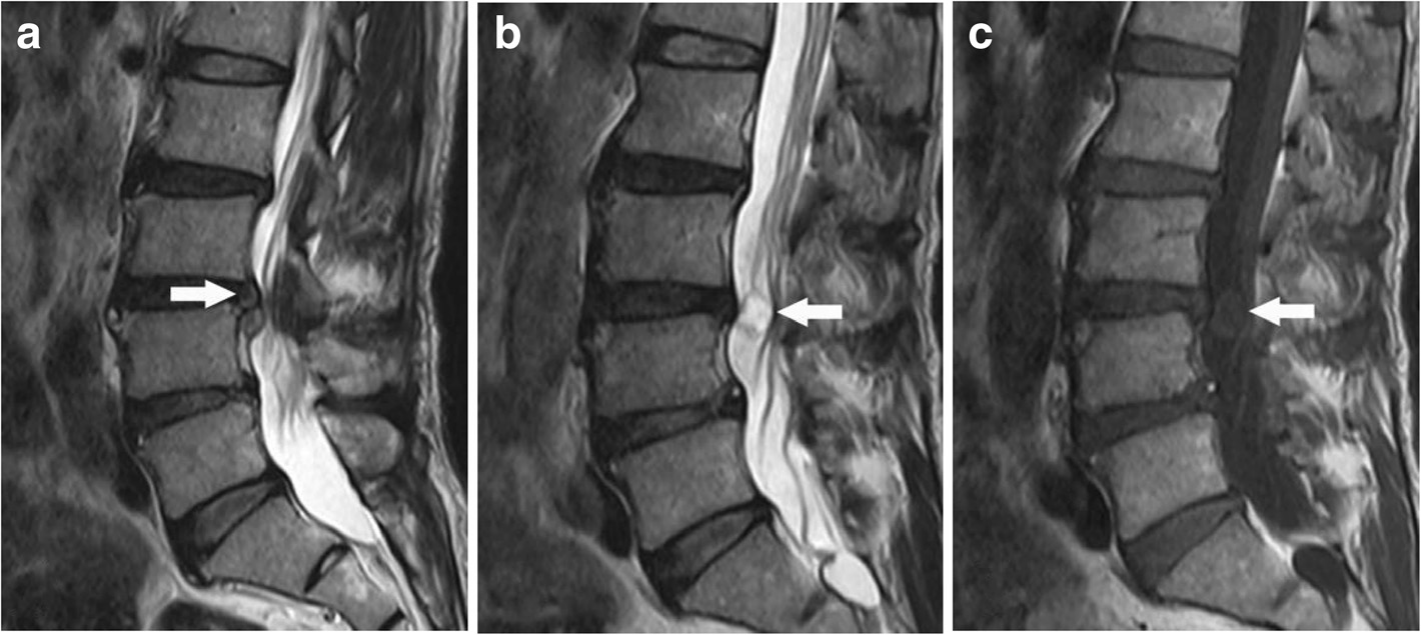
T2-weighted sagittal image (**a**) revealed L3/4 disc herniation which compressed the dural sac from the right side. Two slices away, there was a hyperintense intradural mass at the same level (**b**). T1-weighted sagittal image (**c**) demonstrated a hypointense mass behind the L3/4 intervertebral disc

**Fig. 2:**
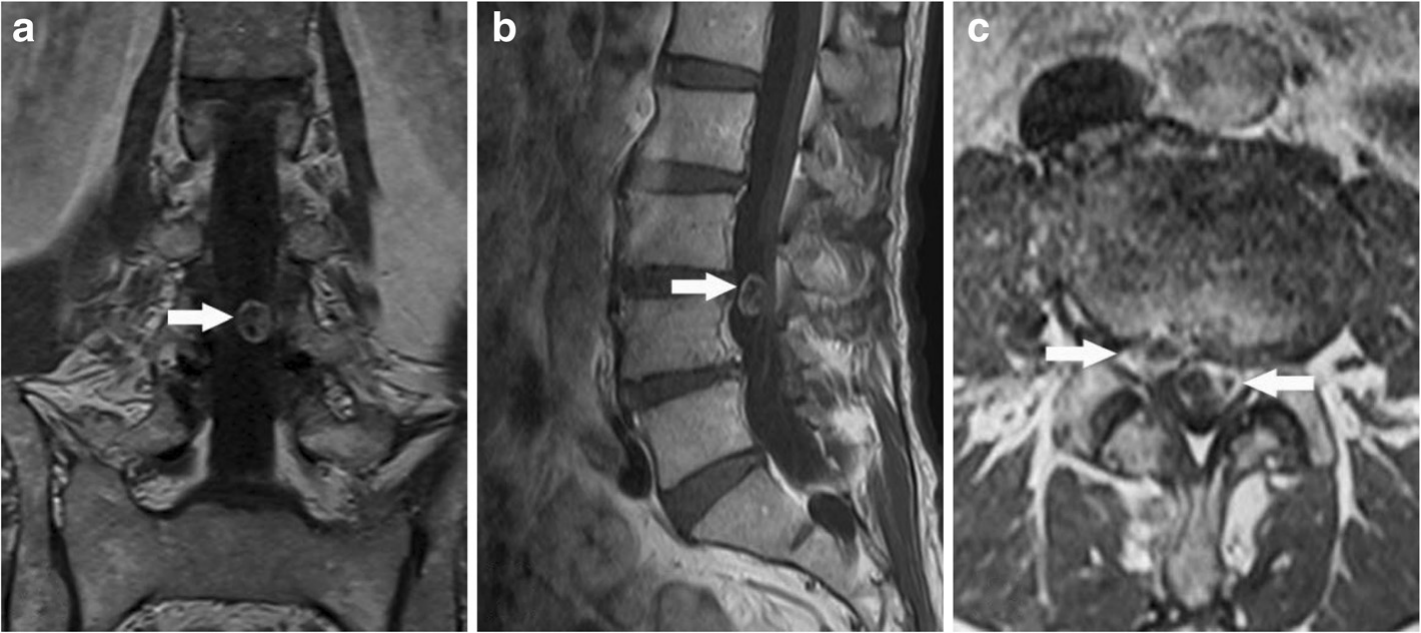
Gadolinium contrast MR images (**a** coronal image, **b** sagittal image) demonstrated a partially enhanced intradural mass at the left side of the L3/4 spinal canal. Axial image (**c**) showed an intradural mass of heterogeneous signal at the left and a herniated disc at the right extradural space

On physical examination, there was mild tenderness on L3-5 spinous processes. While straight leg raising test was negative, femoral stretch test was positive at both sides. Neurological examinations revealed decreased muscle power for the right quadriceps femoris and left tibialis anterior (Manual Muscle Test grade IV). His knee and ankle reflexes at the right leg disappeared, and sensation at the medial side of his left calf diminished. Pathological reflexes were negative at both legs. Lumbar spine MR imaging revealed an intradural lesion (14 × 8 mm^2^) at the left side of the dural sac and a herniated disc of moderate size at the right lateral recess of the L3/4 spinal canal (Figs. 1 and 2).

The patient underwent laminectomy and intrathecal tumor resection. Intraoperatively, the lesion was found to be encapsulated and stuck to a nerve root. After tumor removal, discectomy and posterior lumbar intervertebral fusion were performed. Histological studies revealed a herniated disc and a schwannoma. At the 6-month postoperative follow-up, the patient did not have back and leg pain. Neurological deficits at the extremities fully recovered, and he returned to normal life.

### Discussion

The current case highlights a rare situation that symptomatic disc herniation and spinal tumor present simultaneously in a spinal segment. To our knowledge, there are only four similar cases of lumbar disc herniation coexisting with intradural tumor reported in English literatures [3–6]. Albert et al. reported a case of L4/5 disc herniation presented together with a neurinoma at the L5 nerve root in a 52-year-old man [3]. The clinical manifestations revealed typical signs of L5 root compression from the herniated disc, and the tumor was an accidental finding on myelography. Liu et al. reported a 51-year-old man with L2/3 disc herniation coexisting with a schwannoma causing cauda equina syndrome [4]. The patient had suffered from low back pain for 3 years and was diagnosed with L2/3 lumbar disc herniation. To begin with, his back pain was relieved by conservative treatment but deteriorated progressively till cauda equina syndrome occurred. Bhatia et al. reported a paraganglioma coexisting with L5/S1 disc herniation in a 33-year-old man [5]. The clinical presentation was left sciatica associated with paresthesia in the left S1 dermatome. In 2014, Baek et al. described another case of intradural schwannoma coexisting with lumbar disc herniation at L4/5 disc level in a 71-year-old woman who suffered from lower back pain and L5 radiculopathy [6]. Intrathecal tumor resection and discectomy were performed using a posterior approach in these cases. In our case, right leg radiculopathy resulted from L4 nerve root compression due to L3/4 disc herniation. Some other symptoms, such as claudication and leg weakness, may be the results of both pathologies. We used a single operation to address both conditions, as did others [4–6].

Spinal schwannoma is a benign nerve sheath tumor, which comprises approximately 15 % of all spinal tumors [2]. The vast majority of schwannomas was located at the intradural space and occasionally presented as extradural or a dumbbell-shaped lesion [7]. Intradural schwannoma may produce symptoms similar to those of disc herniation, such as back pain and neurological deficits [8]. On MR images, schwannoma typically presents as an isolated and encapsulated mass, as hypointense or isointense on T1W images and hyperintense on T2W images [2]. Contrast MR imaging may reveal a well-delineated enhanced mass and, thus, is useful for differential schwannoma from disc degeneration [9]. In the present case, gadolinium contrast MR images revealed two heterogeneous signal masses at the same level, with one at the right extradural space and another at the left intradural space. As a result, the establishment of diagnosis was relatively easy.

In general, posterior epidural migration of a herniated lumbar disc fragment, a rare situation of disc herniation, should also be included in the differential diagnosis for extradural and intradural tumors [10–12]. Extradural disc fragment presents signals similar to those of a disc on both T1-weighted and T2-weighted MR sagittal images [11]. Moreover, disc fragment may demonstrate peripheral enhancement on contrast MR images [10, 11]. Even rarer, calcified disc sequestration may mimic an intradural spinal tumor and had MR findings similar to that of schwannoma [13]. Occasionally, a histological study is the only way to verify the diagnosis.

When clinical symptoms cannot be fully explained by an identified pathology, the coexistence of another spinal pathology should be considered. It is easy to establish the diagnoses when a spinal tumor and a herniated disc present in the same spinal region, as both pathologies are displayed on a MR study. It is noteworthy, however, that spinal tumor and lumbar disc herniation may present at different spinal regions and a single MR study may not be able to reveal both. Knafa reported a patient who presented rapidly progressive spinal cord compression following discectomy [14]. Her symptoms temporally restored but soon developed progressing paralysis in her right leg. Retrospective MR revealed an extramedullary tumor at T1/2 level. Another scholar reported a similar case of misdiagnosed thoracic tumor with neurological deficits deteriorated after decompression surgery for lumbar spinal stenosis [15]. Symptoms of upper neuron compression in patients who suffer from lumbar degenerative disorders, therefore, should be carefully examined to exclude possible concomitant pathology in the thoracic or cervical regions.

## Conclusions

We reported a rare case with disc herniation and spinal tumor present at the same spinal level. Due to the similarities of clinical presentations and image findings for spinal tumor and disc herniation, and at an early stage, a diagnosis of spinal tumor was missed in our case. We highlighted the possibilities of concomitant dual or epidural pathologies in some cases. Complete history, careful physical examination, and investigative measures, such as contrast MR imaging, are helpful to establish throughout diagnoses.

### Consent

Written informed consent was obtained from the patient for the publication of this case presentation and accompanying images. A copy of the written consent is available for the review by the Editor-in-Chief of this journal.
